# P02.142. Efficacy of energy therapy in relieving anxiety and pain in patients undergoing lumbar spine fusion surgery

**DOI:** 10.1186/1472-6882-12-S1-P198

**Published:** 2012-06-12

**Authors:** N Cotter, W Dowling, C Gatto, A Gallagher, J Smith, R Evans, R Musanti, R Bustami

**Affiliations:** 1 Division of Integrative Medicine Atlantic Health, Montclair, USA; 2 Department of Orthopedics, Morristown Medical Center, Morristown, USA; 3 Department of Nursing, Morristown Medical Center, Morristown, USA; 4 Division of Integrative Medicine, Morristown Medical Center, Morristown, USA; 5 Department of Research, Atlantic Health, Morristown, USA

## Purpose

Energy therapies in the hospital setting are met with various levels of acceptance due to questions of efficacy and evidence base. This prospective randomized controlled study quantifies the impact of one form of energy therapy on post-operative anxiety and pain in the lumbar spine surgery population.

## Methods

After meeting inclusion and exclusion criteria, patients that consented to participate in the study were randomized into standard post operative care (Control), standard post operative care plus a Healing Touch therapy intervention (Treatment), and standard post operative care plus an attention-control intervention (Sham). Patients were asked to complete the Visual Analog Anxiety Scale, Visual Analog Pain Scale, and Hospital Anxiety Depression Scale (HADS) both prior to and after receiving four consecutive sessions of Healing Touch intervention, attention-controlled intervention, or the standard of care. Baseline data were compared among the three groups using the chi-square test. The Sham and Treatment group were compared in terms of average change (pre vs. post Healing Touch therapy intervention) in HADS, anxiety and pain scores using the t-test.

## Results

A total of 75 patients were included: 25 in the Control group (33%), 24 (32%) and 26 (35%) in the Sham and Treatment groups, respectively. The three groups were similar in terms of baseline factors (p>0.05). Results from comparing the Sham and Treatment group in terms of the change in anxiety and pain scores showed marginally statistically significant differences in anxiety scores in session 2, statistically significant differences in anxiety in session 3, and statistically significant differences in pain scores measured in sessions 2, 3 and 4. No differences were observed between the two groups in terms of the change in HADS (p>0.05).

## Conclusion

Healing Touch demonstrated a statistically significant reduction in post-operative pain and is a viable adjunct in the care of patients undergoing lumbar spine surgery.

